# Supplementary figures and images for: Environmental Drivers of Genetic Divergence in Two Corals From the Florida Keys
Source: Evol Appl. 2025 Jun 29;18(7):e70126. doi: 10.1111/eva.70126 (PMC12206660; doi:10.1111/eva.70126)

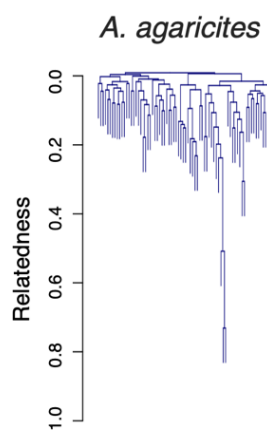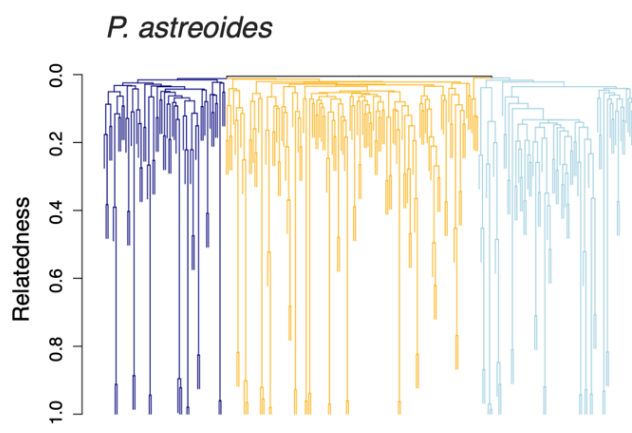

Supplement: Supplementary file 1 — Figure S1. Hierarchical clustering trees based on relatedness. P. astreoides contains visibly more closely related and clonal (relatedness approaching 1) groups. [file EVA-18-e70126-s002.pdf]

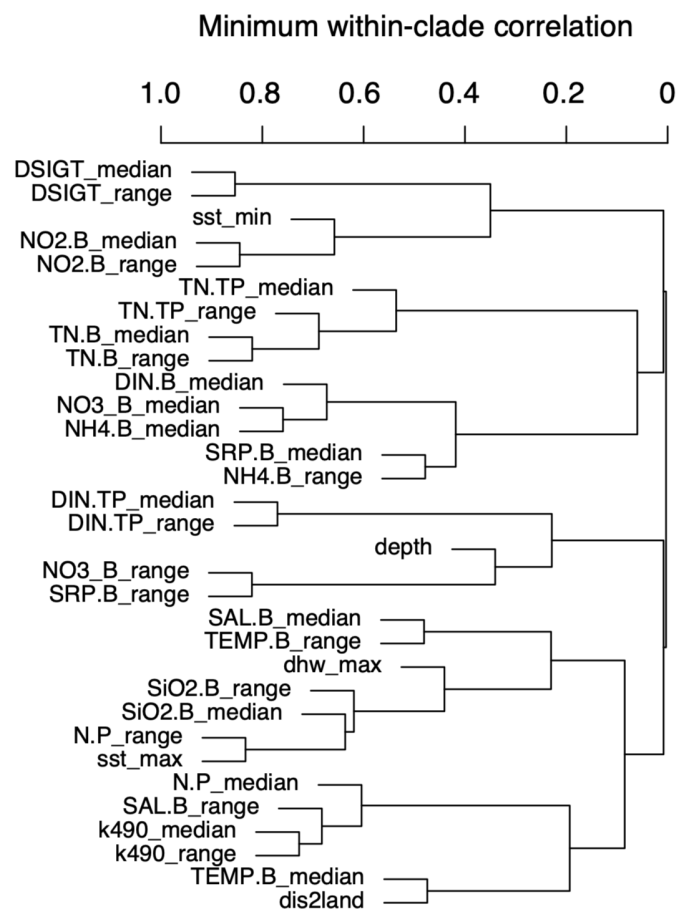

Supplement: Supplementary file 2 — Figure S2. Hierarchical clustering tree of retained environmental predictors after pruning variables that were correlated with r = 0.9 or higher. The tree is based on absolute correlation across sampled sites (complete linkage clustering). [file EVA-18-e70126-s010.pdf]

*A. agaricites*

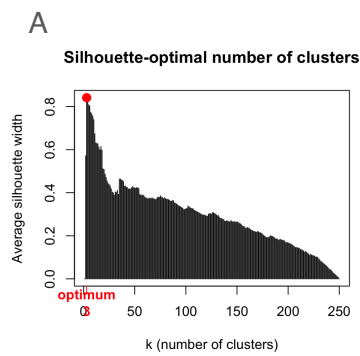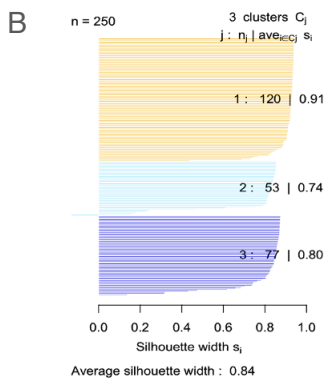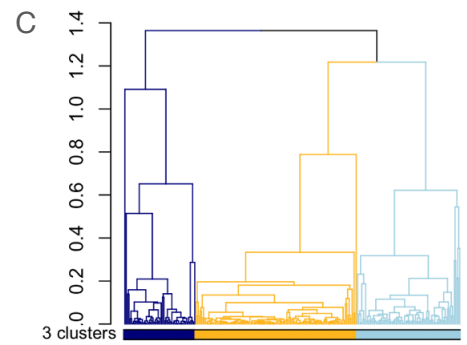

*P. astreoides*

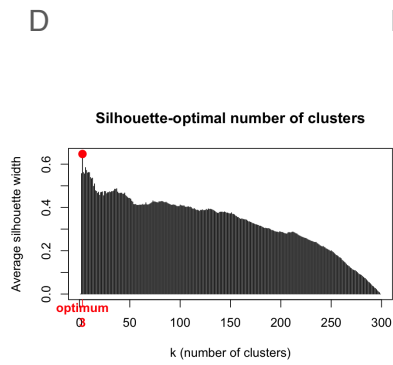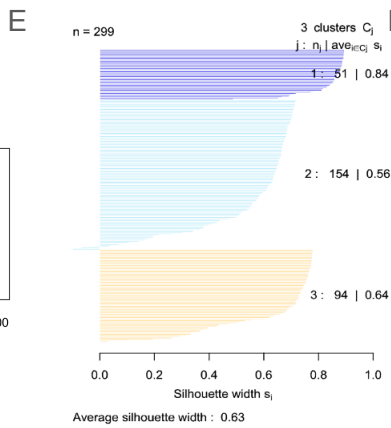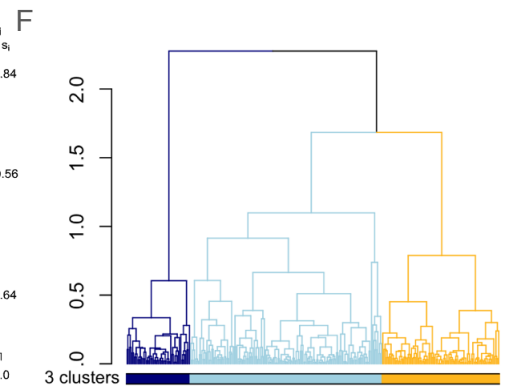

Supplement: Supplementary file 3 — Figure S3. Optimal number of genetic clusters based on silhouette width (measure of the difference between within‐cluster distances and distances to members of the next closest cluster). (A–C): A. agaricites , (D–F): P. astreoides. (A, D) Average silhouette widths for different numbers of clusters (k), based on the UPGMA clustering algorithm. In both cases, the widest silhouettes are observed at k = 3. (B, E) Silhouette widths for individual samples for k = 3. (C, F) Hierarchical clustering trees colored by cluster assignment. [file EVA-18-e70126-s007.pdf]

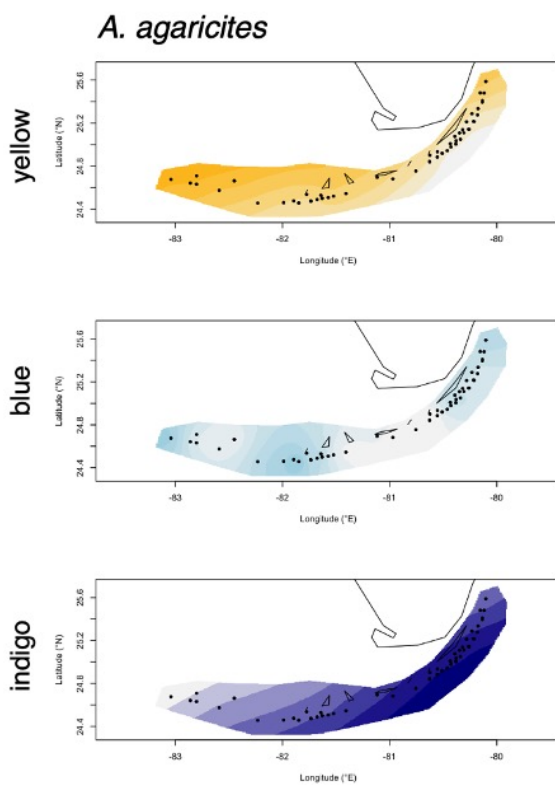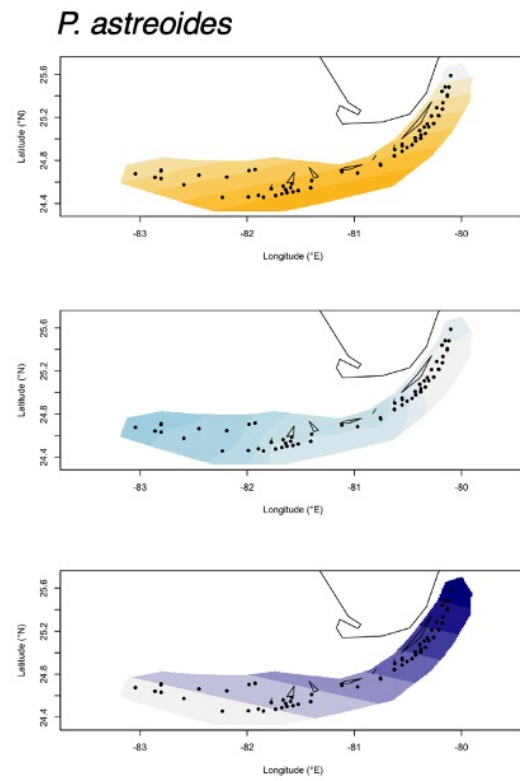

Supplement: Supplementary file 4 — Figure S4. Distribution of genetic lineages of A. agaricites and P. astreoides across the FL Keys Reef Tract (FKRT). Spatial interpolation was performed using Tess3r (version 1.1.0, Caye et al. 2016) using ancestry proportions from Admixture analysis (Skotte et al. 2013). Dots indicate genetic sampling sites and more saturated colors indicate higher proportion of ancestry to each lineage. While distribution of lineage is uneven across the FKRT, they overlap broadly in their distribution. [file EVA-18-e70126-s005.pdf]

yellow

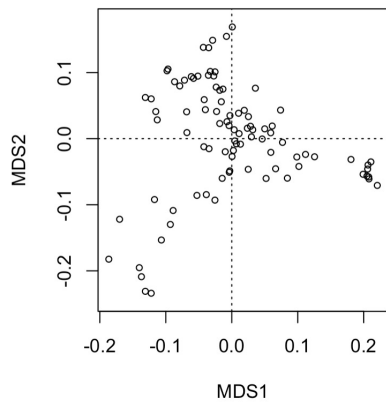

blue

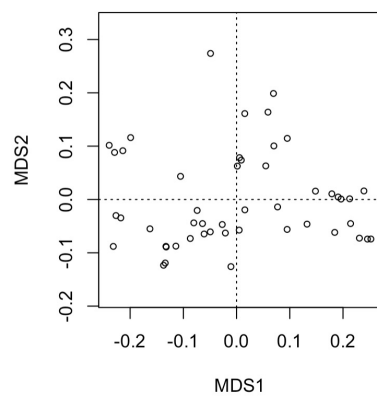

indigo

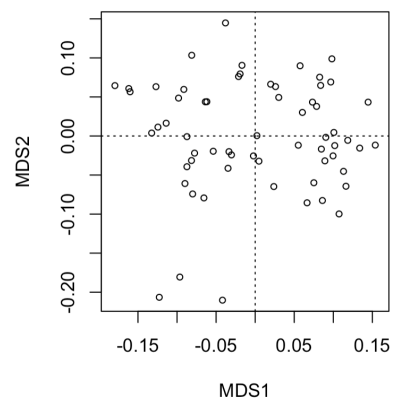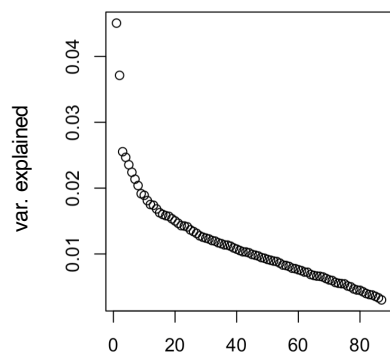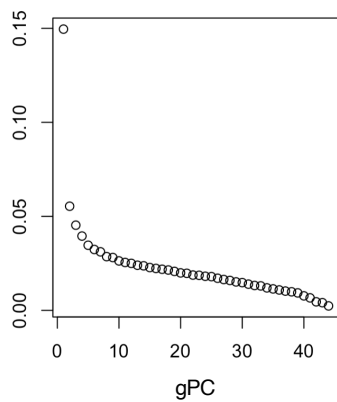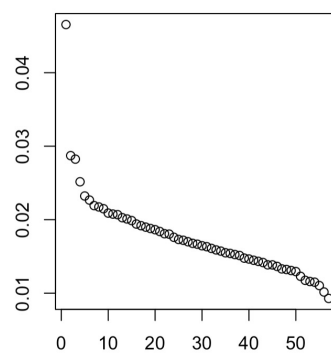

Supplement: Supplementary file 5 — Figure S5. The first two gPCs (top row) and proportion of variation explained by each gPC (bottom row) for three A. agaricites lineages. [file EVA-18-e70126-s003.pdf]

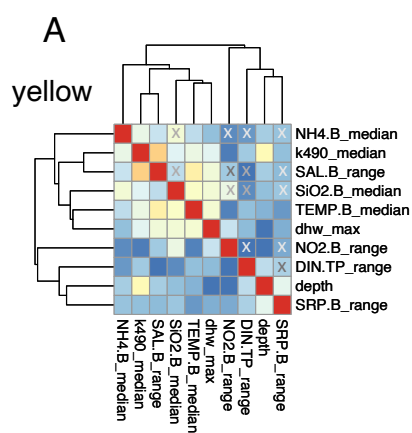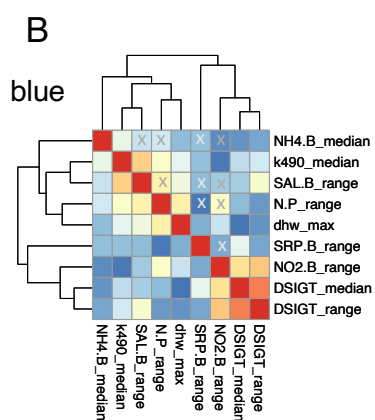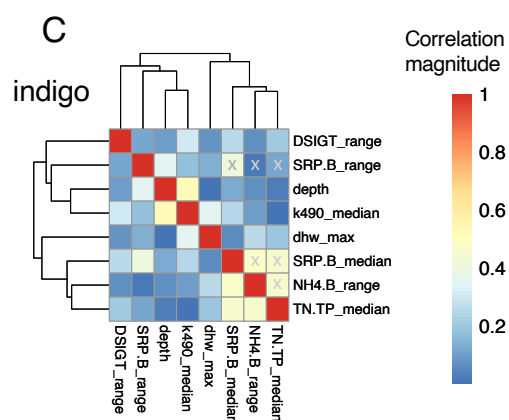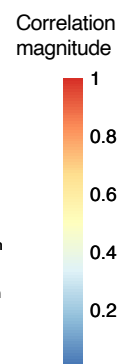

Supplement: Supplementary file 6 — Figure S6. Correlations between retained important predictors for three A. agaricites lineages. Heatmaps show the magnitude (absolute value) of correlation between predictors across coral samples. Cells corresponding to correlations between water chemistry parameters are marked by “x.” [file EVA-18-e70126-s009.pdf]

*A. agaricites*

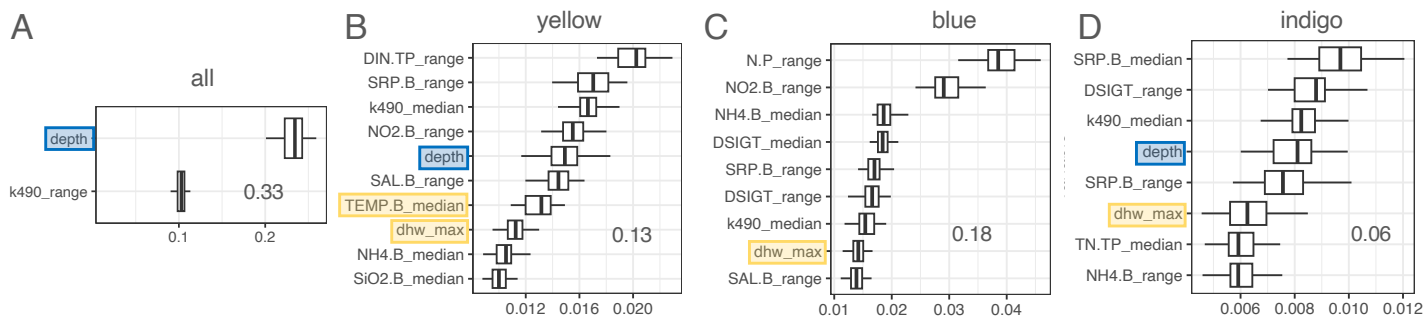

*P. astreoides*

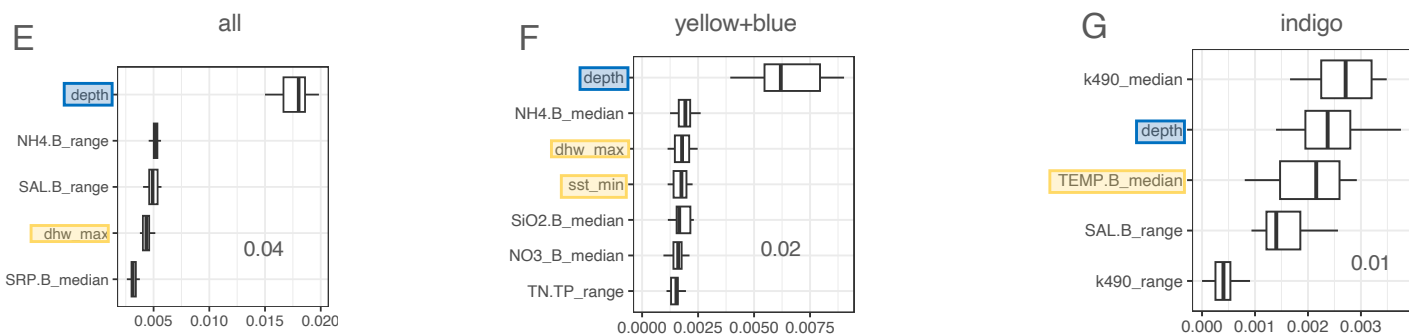

Supplement: Supplementary file 7 — Figure S7. Proportion of total genetic variation explained by predictors retained after mtry‐based selection procedure. (A–D) Agaricia, (E–G) Porites. The boxplots summarize importance (cross‐validation R 2) across 25 ordination jackknife replicates. Depth is labeled by blue rectangles, thermal variables—by yellow rectangles; all other variables describe water chemistry except k490 (turbidity) and DSIGT (water column stratification). Sample set is identified by the text label at the top of each panel: panels A and E show predictions based on all samples of a species, other panels show predictions based on subsets of samples corresponding to cryptic lineages. [file EVA-18-e70126-s006.pdf]

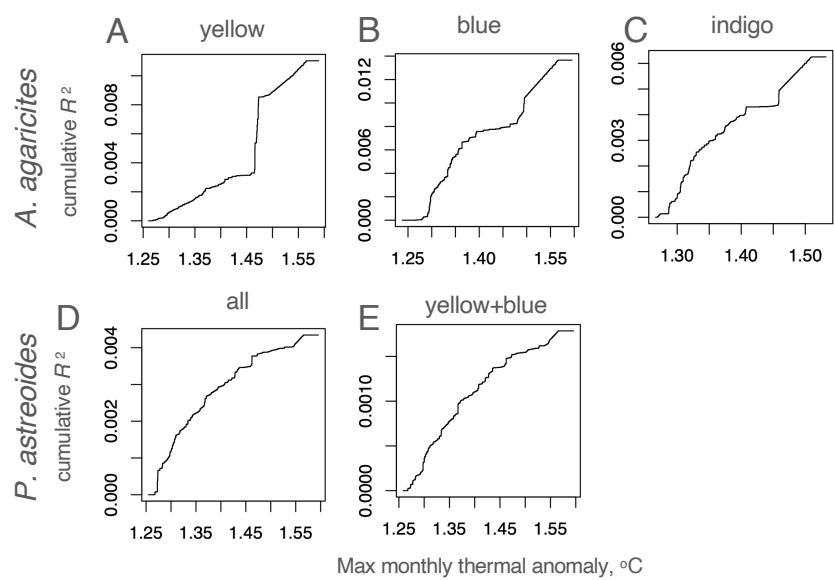

Supplement: Supplementary file 8 — Figure S8. Turnover curves for maximum monthly thermal anomaly (dhw_max). (A–C) Agaricia, (D–E) Porites. Label on top of each panel identifies the sample set: either whole species (D) or cryptic lineages (A–C, E). [file EVA-18-e70126-s011.pdf]

*A. agaricites*

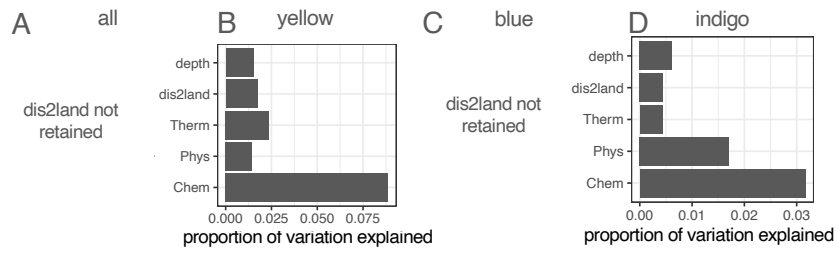

*P. astreoides*

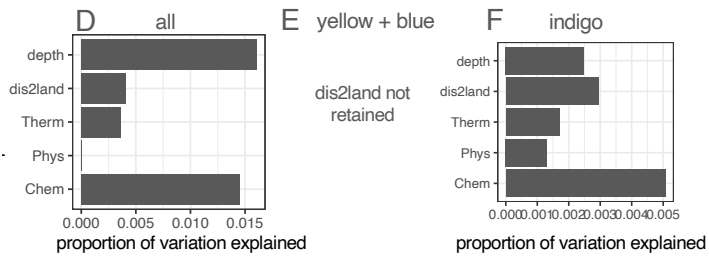

Supplement: Supplementary file 9 — Figure S9. RDAforest models including distance to nearest land (dis2land) retain original predictors and do not substantially differ from original models. This Supplementary Figure hould be compared to Figure 2. Bar charts are missing for analyses where dis2land did not pass the mtry‐based selection criteria. [file EVA-18-e70126-s004.pdf]

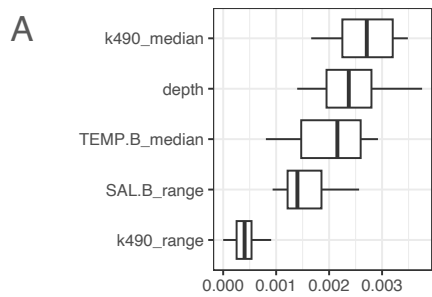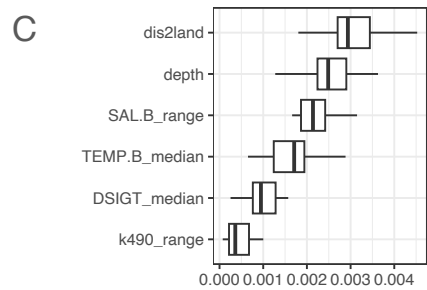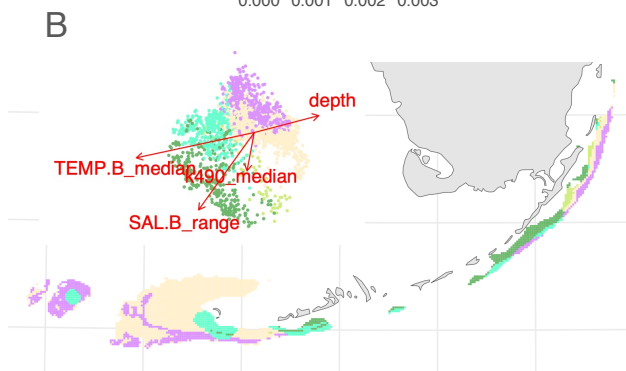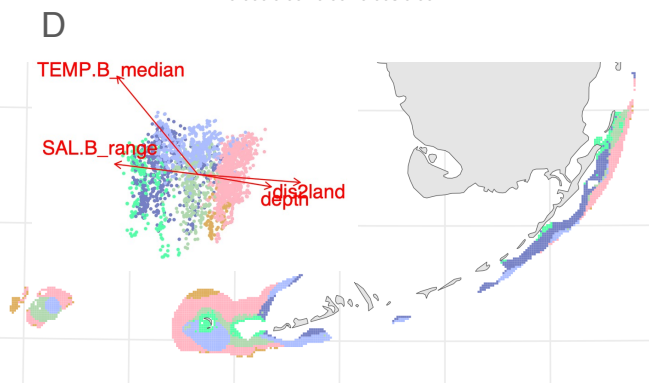

Supplement: Supplementary file 10 — Figure S10. Distance to land does not invalidate specific environmental variables even if it is the most important predictor. Comparing importances of retained predictors (A, C) and maps of adaptive neighborhoods (B, D) for models for P. astreoides indigo either excluding distance to land (dis2land) from predictors (A, B), or including it (C, D). [file EVA-18-e70126-s001.pdf]

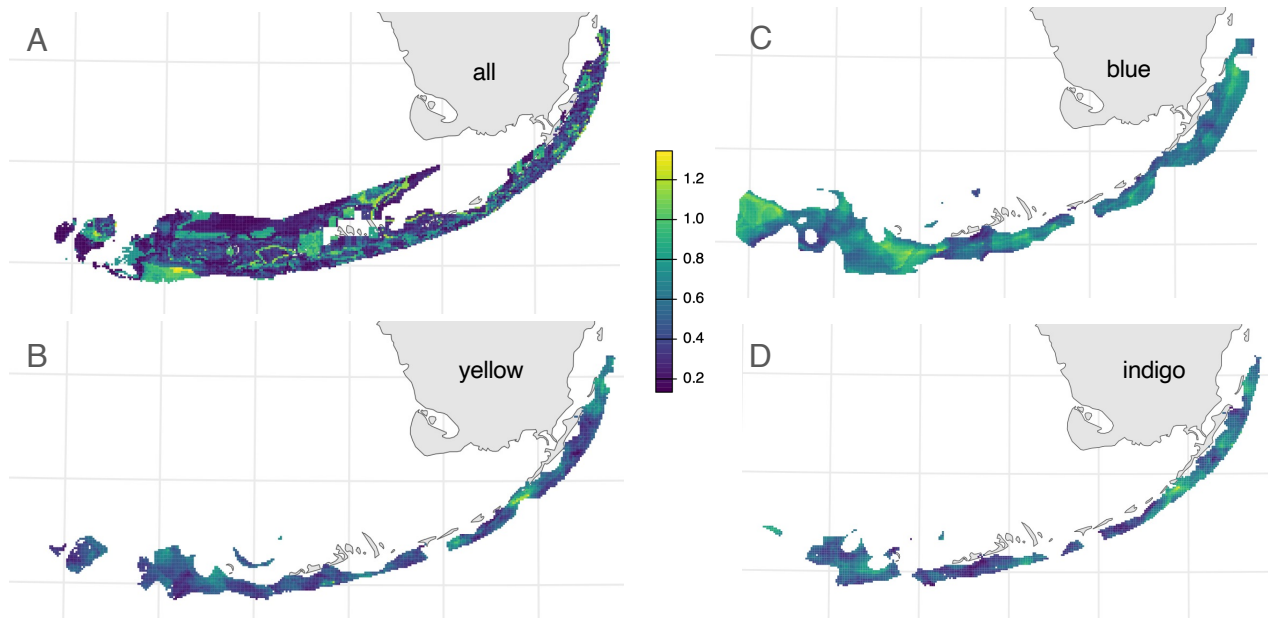

Supplement: Supplementary file 11 — Figure S11. Genetic offsets for A. agaricites , for the whole dataset (A) and for individual cryptic lineages (B–D). [file EVA-18-e70126-s008.pdf]
